# Electron Density Modification of Single Wall Carbon Nanotubes (SWCNT) by Liquid-Phase Molecular Adsorption of Hexaiodobenzene

**DOI:** 10.3390/ma6020535

**Published:** 2013-02-15

**Authors:** Mingxia Lu, Tomonori Ohba, Katsumi Kaneko, Kenji Hata, Motoo Yumura, Sumio Iijima, Hiroto Komatsu, Akira Sakuma, Hirofumi Kanoh

**Affiliations:** 1 Department of Chemistry, Graduate School of Science, Chiba University, Chiba 263-8522, Japan; E-Mails: romeika@pchem2.s.chiba-u.ac.jp (M.L.); ohba@pchem2.s.chiba-u.ac.jp (T.O.); 2 Research Center for Exotic Nanocarbon (JST), Shinshu University, Nagano 380-8553, Japan; E-Mail: kkaneko@shinshu-u.ac.jp; 3 Center of Advanced Carbon Materials, Advanced Industrial Science and Technology, Tsukuba 305-8565, Japan; E-Mails: kenji-hata@aist.go.jp (K.H.); m.yumura@aist.go.jp (M.Y.); s-iijima@aist.go.jp (S.I.); 4 Department of Physics, Meijyo University, Nagoya 468-8502, Japan; 5 Technology Center, Godo Shigen Co. Chosei-mura, Chosei, Chiba 299-4333, Japan; E-Mails: h.komatsu@godoshigen.co.jp (H.K.); sakuma@godoshigen.co.jp (A.S.)

**Keywords:** single wall carbon nanotube, hexaiodobenzene, adsorption, charge transfer

## Abstract

Electron density of single wall carbon nanotubes (SWCNT) is effectively modified by hexaiodobenzene (HIB) molecules using liquid-phase adsorption. UV-Vis-NIR absorption spectra of the HIB-adsorbed SWCNT, especially in the NIR region, showed a disappearance of S_11_ transitions between the V1 valance band and the C1 conduction band of van Hove singularities which can be attributed to the effective charge transfer between HIB and the SWCNT. The adsorption of HIB also caused significant peak-shifts (lower frequency shift around 170 cm^−1^ and higher shift around 186 cm^−1^) and an intensity change (around 100–150 cm^−1^ and 270–290 cm^−1^) in the radial breathing mode of Raman spectra. The charge transfer from SWCNT to HIB was further confirmed by the change in the C1s peak of X-ray photoelectron spectrum, revealing the oxidation of carbon in SWCNT upon HIB adsorption.

## 1. Introduction

Since their discovery [1], single wall carbon nanotubes (SWCNT) have attracted considerable attention in widely diverse fields owing to their remarkable mechanical, thermal and electrical properties [2,3,4]. More recently, studies on the electrical properties of SWCNT have focused on the charge transfer interaction of SWCNT with electron donor or acceptor molecules that allow the manipulation of electrical conductivity of SWCNT [5,6,7]. Conventional modification of SWCNT has been realized by intercalation with iodine or halogenides of Na, K, Rb, Cs, Ca, Cu, and Ag through gas phase doping or liquid phase adsorption methods [8,9,10,11,12,13]. These SWCNT modified with metal halogenides always show a p-type behavior, which means that electrons are always withdrawn from the valence band of SWCNT to the adsorbates and the main charge carrier in the SWCNT are holes. However, this empirical rule was disproved by Jung *et al.* [14], who observed a slight down-shift of a C1s peak in the X-ray photoelectron spectra upon iodine intercalation, indicating that iodine can act as a weak electron donor for SWCNT. They also proved that the iodine has a partial positively charged state of I^+0.08−0.1^ by I LI-edge X-ray absorption near-edge structure (XANES) analysis. Hayakawa *et al.* [8] reported that the adsorption of iodine also leads to a dramatic enhancement in the conductivity of SWCNT. Hexaiodobenzene (HIB, C_6_I_6_) molecules exhibit a two-electron oxidation that generates a di-cation (C_6_I_6_)^2+^ from HIB (C_6_I_6_) [15]. In addition, (C_6_I_6_)^2+^ shows σ-aromaticity co-existing with the conventional π-aromaticity that it shares with its neutral parent [16]. Because of the unique electronic structure of HIB, in this study, SWCNT were modified by HIB adsorption through a liquid-phase adsorption method. Spectroscopic techniques, such as UV-Vis-NIR adsorption, Raman, and X-ray photoelectron spectroscopy, were employed to understand the electron-density changes of SWCNT after the HIB adsorption. Quantitative analysis of the adsorbed amounts was also carried out by a thermogravimetric method.

## 2. Results and Discussion

**Figure 1:**
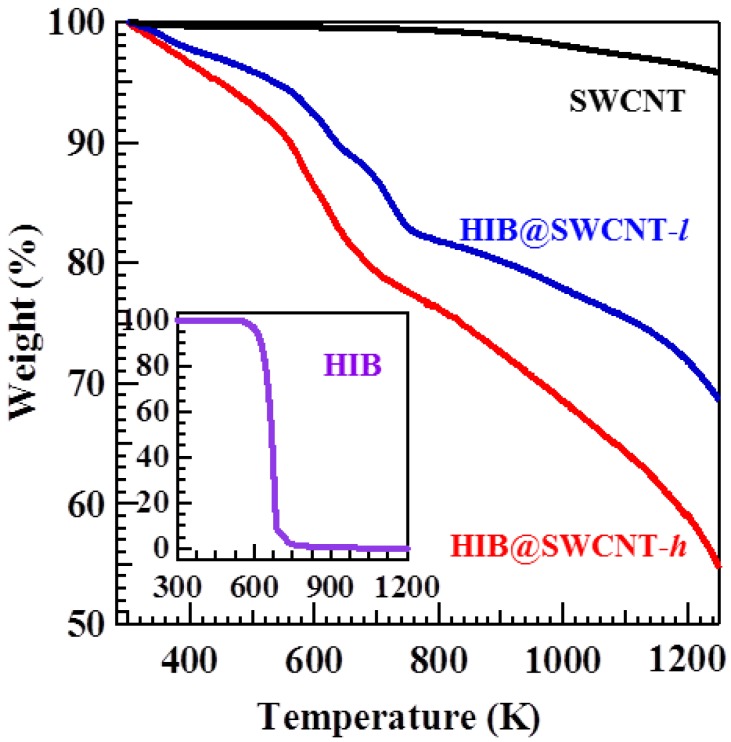
Thermogravimetric analysis (TGA) curves of hexaiodobenzene (HIB) (inset), single wall carbon nanotubes (SWCNT), HIB-adsorbed SWCNT with different HIB concentrations of 46.2 mg L^−1^ and 127.2 mg L^−1^, are denoted as HIB@SWCNT-*l* and HIB@SWCNT-*h*, respectively.

A TGA provides a quantitative way to determine the thermal stability of SWCNT as well as the amount of HIB molecules adsorbed on SWCNT. The TGA curves of HIB, SWCNT, HIB@SWCNT-*l* (46.2 mg L^−1^), and HIB@SWCNT-*h* (127.2 mg L^−1^) were measured under N_2_ environment as shown in Figure 1. The TGA curve of SWCNT shows only 0.8% weight loss until 800 K. This indicates the high purity and high thermal stability of SWCNT. The inset graph shows that the weight loss of HIB starts from 520 K, and it is complete at 850 K, which corresponds to the decomposition of HIB. Therefore, the weight loss ranging from 520 to 850 K in the TGA curve of HIB-adsorbed SWCNT can be attributed to the decomposition of the adsorbed HIB. In this range the weight loss is about 15.0 wt % of the total mass for HIB@SWCNT-*l* and 18.6 wt % for HIB@SWCNT-*h*, respectively. The amount of adsorbed HIB per gram can be calculated by the following equation:

(1) $$\text{Adsorbed amount}=\frac{m_{\text{HIB}}}{m_{\text{SWCNT}}}=\frac{\text{wt}\;\%\text{ of HIB}}{1-\text{wt}\;\%\text{ of HIB}}\times 1000\;\left(\frac{\text{mg}}{\text{g}}\right)$$

where $m_{HIB}$ is the mass of HIB adsorbed on SWCNT, $m_{\text{SWCNT}}$ is the mass of SWCNT, and wt % is the weight loss. By using this equation, the calculated amount of adsorbed HIB is about 176.5 mg g^−1^ for HIB@SWCNT-*l* and 228.5 mg g^−1^ for HIB@SWCNT-*h*.

**Figure 2:**
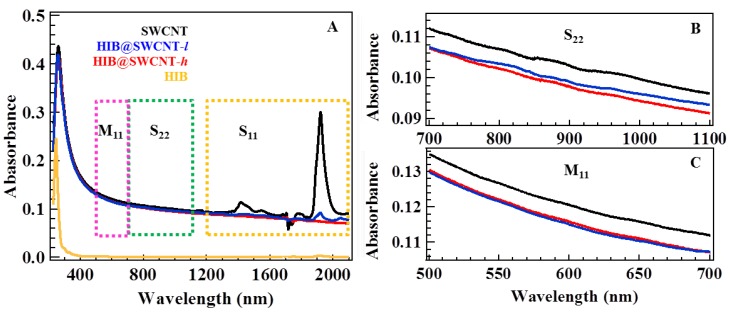
UV-Vis-NIR absorption spectra of SWCNT, HIB@SWCNT-*l*, HIB@SWCNT-*h* and HIB (**A**); the expanded spectra at S_22_ (**B**) and M_11_ (**C**).

Figure 2 shows the UV-Vis-NIR absorption spectra of SWCNT, HIB@SWCNT-*l*, and HIB@SWCNT-*h*. The UV-Vis-NIR spectrum of SWCNT has the absorption bands in the range of 1200–2100 nm (S_11_), 700–1100 nm (S_22_), and 500–700 nm (M_11_), originating from the interband electronic transitions of van Hove singularities in semiconducting and metallic SWCNT [17].

The change in S_11_ transition after the HIB adsorption indicates the charge transfer between HIB and SWCNT. Furthermore, it should be noted that the intensities of these peaks decrease with increasing the adsorbed amount of HIB and they almost disappear with higher adsorption. This effect can be interpreted either as the withdrawal of electrons from the valence band (*i.e.*, *p*-type doping) or as the injection of electrons to the conductance band (*i.e.*, *n*-type doping) in semiconducting SWCNT [10]. However, the direction of electron transfer cannot be determined only from the absorption spectra since it depends on the band edge alignment between SWCNT and the redox potential of HIB molecules. The slight decrease of peak intensity of M_11_ indicates the weak interaction between metallic SWCNT and adsorbed HIB.

**Figure 3:**
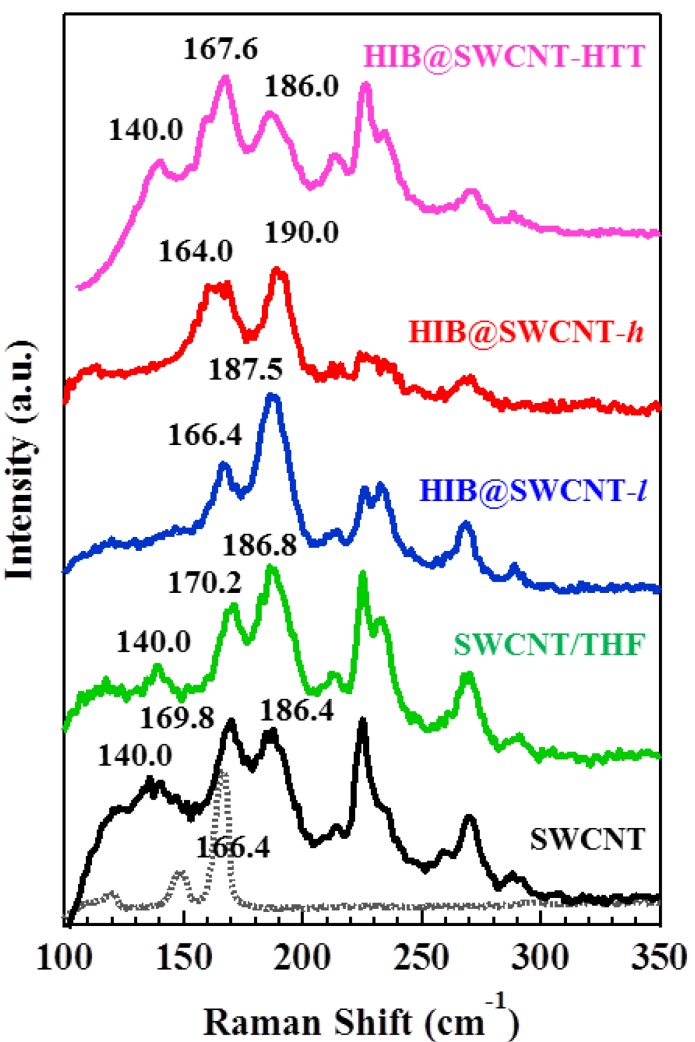
Raman spectra at radial breathing mode (RBM) region of SWCNT, SWCNT/THF without HIB addition, HIB@SWCNT-*l*, HIB@SWCNT-*h*, and HIB@SWCNT-HTT. The spectrum of solid HIB (dash line) is shown as a reference. Here THF is tetrahydrofuran; HTT is high-temperature treatment.

Figure 3 shows the Raman spectra of SWCNT, HIB@SWCNT-*l*, HIB@SWCNT-*h,* and HIB@SWCNT-HTT at the radial breathing mode (RBM) region. In liquid-phase adsorption, the influence of the solvent cannot be neglected. In order to examine the contributions of HIB or THF to the Raman spectrum, the spectrum of SWCNT/THF (prepared by dispersing an identical amount of SWCNT in THF) is also shown in Figure 3 (green line). The peak at 169.8 cm^−1^ shifted to the lower frequency side after the adsorption of HIB: The observed shift was by 3.4 cm^−1^ for HIB@SWCNT-*l* and 5.8 cm^−1^ for HIB@SWCNT-*h*. Further, a significant decrease in the intensities of all the other peaks (from 250 to 300 cm^−1^ and from 100 to 150 cm^−1^) was observed: in particular, the peaks at 140 cm^−1^ disappeared completely. However, SWCNT/THF shows no significant changes in this region. This indicates that the changes in the electronic density of SWCNT can be attributed to the HIB adsorption but not to THF, and it is also further confirmed by HIB@SWCNT-HTT. In the Raman spectrum of HIB@SWCNT-HTT, all peaks are restored to their original state after the removal of HIB molecules from SWCNT by a heat treatment. The disappearance of the RBM peak at 140.0 cm^−1^, suggests a loss of resonance from the nanotubes with a diameter of 1.74 nm (at 140.0 cm^−1^, calculated by $\omega_{\text{HIB}}=232/d+6.5$ [18]) upon the adsorption of a charge transfer molecule into SWCNT. This also testifies to a structural deformation of the SWCNT caused by the contraction of the SWCNT upon crystallization of HIB inside the tubes [19]. This result provides evidence for the effective charge transfer between SWCNT and HIB, and also suggests that the effect of HIB adsorption on the electronic structure is dependent on the chirality of SWCNT.

It should be noted that the Raman spectra of HIB-adsorbed SWCNT are shifted both towards higher and lower frequencies. In detail, the small blue-shifts of the peak at 169.8 cm^−1^ (by 3.4 cm^−1^ for HIB@SWCNT-*l*, and 5.8 cm^−1^ for HIB@SWCNT-*h*) should be caused by a convolution of the peak at 169.8 cm^−1^ of SWCNT and 166.4 cm^−1^ of HIB. On the other hand, the peak at 186.4 cm^−1^ displays a red-shift upon the HIB adsorption, which points to the hardening of radial breathing motion of carbon atoms in SWCNT with a diameter of 1.29 nm [12,20]. The origin of this shift can be attributed either to an increase in C–C binding energy due to the charge transfer from SWCNT to HIB or, probably, to the mechanical hindrance such as the hardening and stiffening of C–C bonds [12,21,22]. From the TEM images of HIB-adsorbed SWCNT shown in Figure S1, we found that HIB molecules are distributed inside the tubes, but the binding states and orientation of HIB molecules adsorbed on SWCNT are difficult to resolve, because of the limited resolution.

**Figure 4:**
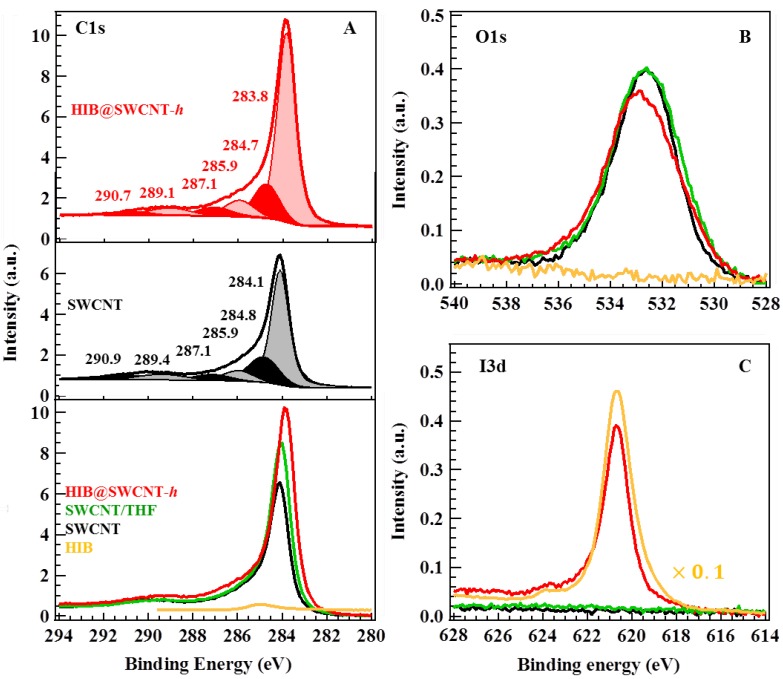
X-ray photoelectron spectra (XPS) spectra of the binding energy of C1s (**A**); O1s (**B**); and I3d (**C**) of HIB (yellow), SWCNT (black), SWCNT/THF (green), and HIB@SWCNT-*h* (red). The sub-peaks in each spectrum were obtained from peak fitting results by using a Lorentzian/Gaussian function.

Systematic XPS analyses were performed to understand the direction of charge transfer between SWCNT and HIB molecules. Figure 4 shows the C1s, O1s, and I3d XPS spectra of HIB, SWCNT, SWCNT/THF, and HIB@SWCNT-*h*. Peak fitting involving a combined Lorentzian and Gaussian function was performed to give better understanding on the origins of binding energies (the details are shown in Table S1). The most intense C1s peak at around 284.1 eV (Figure 4A) is comparable to the C1s binding energy of graphite and is assigned to sp^2^-hybridized carbons on the tube walls. A smaller speak at around 284.8 eV is related to sp^3^-hybridized carbons and may originate from the presence of defects on the tube walls [23]. The other small peaks around 285.9, 287.0, 289.5 and 290.8 eV are assigned to C–O, C=O, O–C=O and π–π* plasmon, respectively [24,25,26]. The sp^2^ peak shows an obvious shift by 0.3 eV towards the lower binding energy side (from 284.1 to 283.8 eV) upon the HIB adsorption, evidencing the charge transfer between SWCNT and HIB. The downshift of the binding energy, consistent with the shift in Fermi level toward the valence band edge, can be interpreted as evidence of the decrease in the electron density in the SWCNT with adsorbed HIB, indicating a charge transfer from SWCNT to HIB. A similar behavior was reported by Mistry *et al.* [27] for nitric-acid-treated SWCNT, and Eliseev *et al.* [12] for AgX doped SWCNT. In the O1s XPS spectra in Figure 4B, the slight shift in the peak position and the small change in the peak shape of the SWCNT upon HIB adsorption indicate that the charge transfer interaction takes place not only between the HIB and π-electron system of SWCNT, but also between the iodine of HIB and the oxygen on the SWCNT, which formed in the oxidation process for the removal of tube caps.

## 3. Experimental Section

Super-growth SWCNT were synthesized by a chemical vapor deposition (CVD) process (Center of Advanced Carbon Materials, AIST). The SWCNT samples were used without further purification because of their high carbon purity (higher than 99.98%) [28]. Tube caps of as-prepared SWCNT were removed by oxidation at 773 K under Ar and O_2_ mixed gases for 1 h. For the preparation of HIB-adsorbed SWCNT, a typical procedure was as follows: 3 mg of SWCNT were dispersed in 50 mL tetrahydrofuran (THF) solution by ultrasonication using an ultrasonic cleaner (FU-50C, 28 kHz) at 298 K for 2 days. Afterwards, hexaiodobenzene (HIB) (2.31 mg and 6.36 mg) was added to the SWCNT dispersion, and then the mixture was further dispersed by sonication for 15 min. The samples were denoted as HIB@SWCNT-*l* and HIB@SWCNT-*h*, respectively.

Then, the mixture was moved into a water bath, and kept at the temperature of 298 K for 1 week to reach the adsorption equilibrium. After filtration, the remaining solid was washed with THF to remove the free HIB molecules and was dried under vacuum at 373 K overnight. After the analysis, a HIB@SWCNT-*h* sample was further heat-treated up to 1273 K at a ramp rate of 5 K min^−1^ under N_2_ at a flow rate of 100 cm^3^ min^−1^. This sample was denoted as HIB@SWCNT-HTT.

Thermogravimetric analysis (TGA) was performed on a thermogravimetric analyzer (Shimadzu; DTG-60AH) at a heating rate of 5 K min^−1^ and N_2_ flow rate of 100 cm^3^ min^−1^. The changes in the electronic properties of SWCNT upon HIB adsorption were measured through the following methods. Raman spectra of each sample were measured on a dried solid by Raman spectrometer (JASCO; NRS-3100) with the excitation wavelengths of 532 nm (power 0.1 mW). X-ray photoelectron spectra (XPS) were measured with X-ray photoelectron spectrometer (JEOL; JPS-9010MX) using monochromatized MgKα radiation as a photon source. The optical absorption spectra measurement (UV-Vis-NIR spectrophotometer, JASCO, V-670) was performed on an HIB-adsorbed SWCNT solution which was prepared by dispersing HIB-adsorbed SWCNT in THF (20 mg L^−1^) by ultrasonicated for 24 h. High-resolution transmission electron microscope (HRTEM; JEOL, JEM-2100F) observation was carried out by drop-casting dispersed solution of HIB-adsorbed SWCNT onto carbon-film-supported copper grids.

## 4. Conclusions

By using liquid-phase molecular adsorption of HIB to SWCNT, the electronic structure of SWCNT could be modified. The electronic structure changes of SWCNT were investigated by spectroscopic methods. UV-Vis-NIR absorption spectra analysis showed that the interaction of HIB could induce a change in the electron density of state in SWCNT. The disappearance of the RBM-peak at 140 cm^−1^ and an up-shift in the RBM-band at 186.4 cm^−1^ provide evidence for the effective charge transfer from SWCNT to HIB. The slight downshift of C1s XPS peak revealed a slight oxidation of carbon in SWCNT upon HIB adsorption. From these results, we conclude that HIB act as an electron acceptor for SWCNT.

## Supplementary materials

Supplementary File 1
