# Supplementary material for: Electron Density Modification of Single Wall Carbon Nanotubes (SWCNT) by Liquid-Phase Molecular Adsorption of Hexaiodobenzene
Source: Materials (Basel). 2013 Feb 15;6(2):535–43. doi: 10.3390/ma6020535 (PMC5452087; doi:10.3390/ma6020535)
Supplement: Supplementary File 1 [file materials-06-00535-s001.pdf]

Supporting Information

Figure S1. TEM image of as-prepared HIB-adsorbed SWCNT. An illustration of HIB molecule with the diameter assumes that HIB molecules are distributed inside the tubes as shown in Figure S1-E.

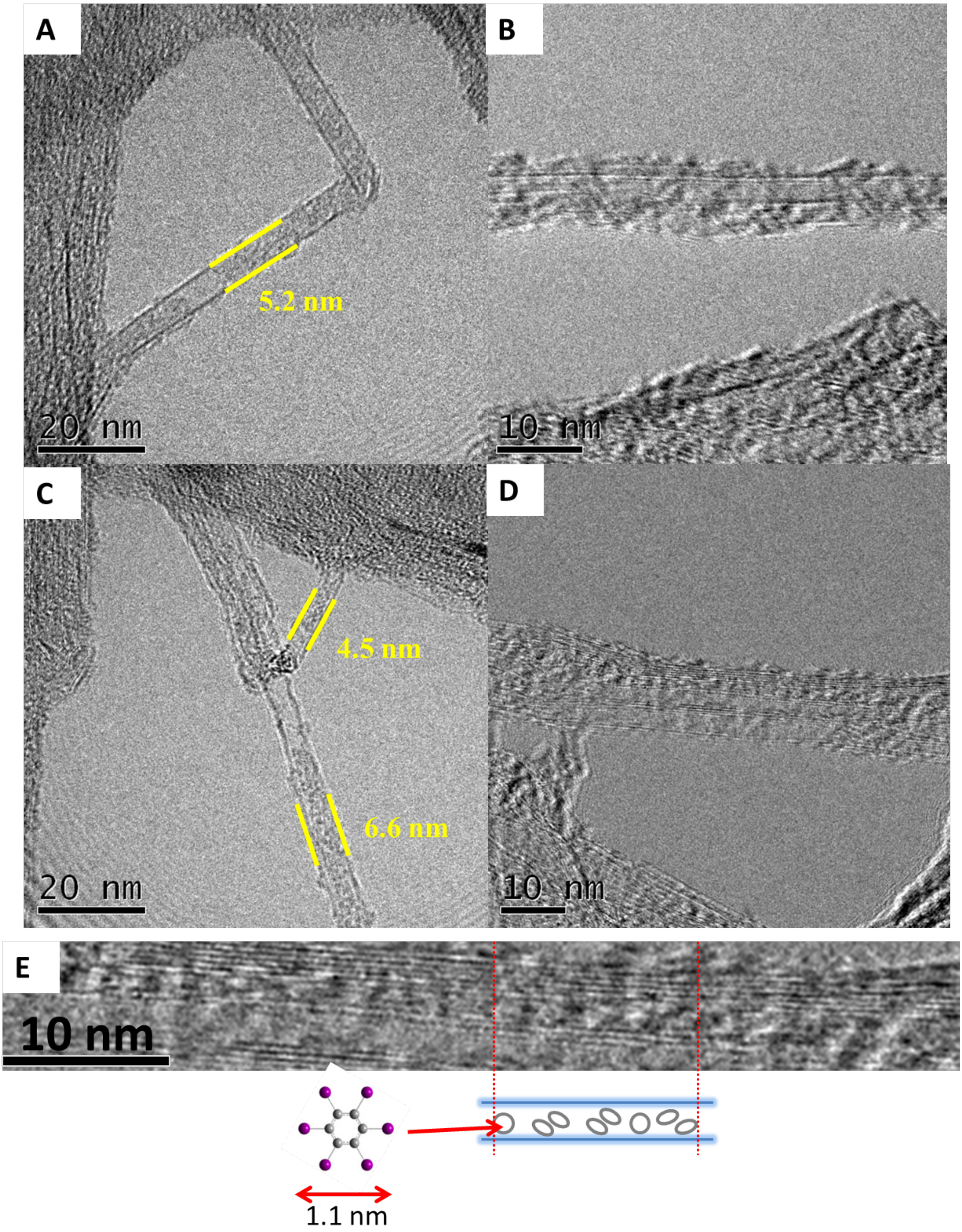

Table S1. The multi-peak fitting parameters of XPS C1s spectra

Sample	Position (eV)	Area	FWHM (eV)
SWCNT	284.1	691.4	0.95
	284.8	212.6	1.59
	285.9	85.5	1.56
	287.1	58.5	1.88
	289.4	53.0	2.62
	290.9	69.8	2.37
HIB@SWCNT-*h*	283.8	1147.8	0.99
	284.7	246.8	1.38
	285.9	128.4	1.50
	287.1	67.9	1.65
	289.1	80.7	2.12
	290.7	29.7	1.76

© 2013 by the authors; licensee MDPI, Basel, Switzerland. This article is an open access article distributed under the terms and conditions of the Creative Commons Attribution license (http://creativecommons.org/licenses/by/3.0/).
